# Establishment and application of a dual RPA-LFD rapid detection method for *Salmonella* Pullorum and *Salmonella* Enteritidis

**DOI:** 10.1371/journal.pone.0336423

**Published:** 2025-11-19

**Authors:** Can Wang, Tingting Zeng, Houxun Ya, Lijun Wan, Ming Yan, Sisi Luo, Meng Li, Yanfei Deng, Hongyu Ren, Zuoxin Chen, Zhixun Xie, Liji Xie

**Affiliations:** 1 Guangxi Key Laboratory of Veterinary Biotechnology, Guangxi Veterinary Research Institute, Nanning, China; 2 Guangxi Key Laboratory of Animal Breeding and Disease Control, College of Animal Science and Technology, Guangxi University, Nanning, China; Nitte University, INDIA

## Abstract

*Salmonella* species are known to cause a significant decline in poultry production performance and to contaminate various stages of the breeding process, with *Salmonella* Pullorum (*S*. Pullorum) and *Salmonella* Enteritidis (*S*. Enteritidis) being the predominant serotypes responsible for infection in poultry. For rapid diagnosis at an early stage, we developed a method involving dual recombinase polymerase amplification (RPA) combined with a lateral flow dipstick (LFD) in this study, and primers were designed to target the *traJ* gene of *S*. Pullorum and the *Sdf Ⅰ* gene of *S*. Enteritidis. The primers and probes were screened, and the reaction conditions were optimized. The results showed that dual RPA successfully amplified *S*. Pullorum and *S*. Enteritidis DNA within 15 min at 37°C, and when combined with LFD, the entire process (amplification and detection) was completed within 20 min. The detection limits for *S*. Pullorum and *S*. Enteritidis were 1.56 × 10^2^ CFU/mL and 1.38 × 10^2^ CFU/mL, respectively. The developed dual RPA-LFD method specifically targets *S*. Pullorum and *S*. Enteritidis and exhibits no cross-reactivity with other common pathogenic microorganisms. The results for the clinical samples were fully consistent with those obtained using the Bacteriological Analytical Manual (BAM) method. The results of this study demonstrated that the developed dual RPA-LFD method is simple, rapid, specific, and highly sensitive for the simultaneous visual detection of *S*. Pullorum and *S*. Enteritidis, providing a technical reference for primary veterinary laboratories and veterinary field tests.

## Introduction

*Salmonella* species are important food-borne pathogens with a high incidence rate and mortality. They can infect a variety of animals and spread from animals to humans, causing considerable economic losses and public health security problems [1]. According to a previous report, approximately 100 million people are infected with *Salmonella* every year, and 155 000 deaths occur worldwide [2]. Therefore, *Salmonella* has long been a focus of research in the field of bacteriology, both in China and internationally. To date, more than 2 600 *Salmonella* serovars have been identified globally, and more than 250 have been discovered in China [3,4]. *Salmonella enterica* serovar Gallinarum biovar Pullorum (*S*. Pullorum) and *Salmonella enterica* serovar Enteritidis (*S*. Enteritidis) are the most prevalent serovars that infect poultry [5,6]. Pullorum disease is a World Organisation for Animal Health (OIE)-listed poultry disease caused by *S*. Pullorum and is characterized by white viscous diarrhea and acute septicemia in chicks [6]. After infection with *S*. Pullorum, some infected chicks can recover from pullorum disease, and some adult chickens may not show clinical disease symptoms; subsequently, they become a repository of *S*. Pullorum, contaminating other healthy chickens through horizontal and vertical transmission [7]. *S*. Enteritidis has numerous hosts and is known not only to infect poultry but also to act as a potential agent of human gastroenteritis [8]. Chickens infected with *S*. Enteritidis exhibit no obvious clinical symptoms, but *S*. Enteritidis can infect humans through contaminated poultry meat and eggs, causing food poisoning [9]. Therefore, preventing and controlling *S*. Pullorum and *S*. Enteritidis contamination are crucial for protecting the health of chicken breeds and ensuring human safety, for which timely detection is key.

At present, *Salmonella* is detected mainly via traditional laboratory methods [2]. Biochemical tests and slide agglutination tests are used to identify the serovars and isolate strains of *Salmonella*. However, these methods are complex and time-consuming, making it difficult to process the large quantities of samples currently needed for rapid and accurate detection [10]. In recent years, with the development of nucleic acid detection technology, researchers have established a series of rapid and specific molecular biological diagnostic methods, such as polymerase chain reaction (PCR) [11], real-time fluorescence quantitative PCR (qPCR) [12], multiplex PCR [13] and loop-mediated isothermal amplification (LAMP) [2], but these methods also require expensive experimental equipment and trained personnel. Furthermore, the ability to implement these methods is limited in resource-limited settings or for onsite detection. Therefore, a simple, rapid, cost-effective, specific, and sensitive method is crucial for the onsite detection of *Salmonella* to control contamination.

Recombinase polymerase amplification (RPA) is a multienzyme isothermal gene amplification technique known for its simple reaction system. In RPA, a recombinase and primers are allowed to form a recombinase‒primer complex. The complex then scans the double-stranded DNA to locate and bind to its homologous target sequence. Upon binding, the DNA strand is displaced and stabilized by single-stranded binding (SSB) proteins, which creates a starting site for DNA polymerase to initiate rapid amplification [14,15]. The RPA amplification products can be visualized simply by a specific lateral flow dipstick (LFD), which is fast and convenient and allows rapid detection in the onsite laboratory [16]. RPA has been successfully applied in the detection of various pathogenic microorganisms, but there have been no reports on the simultaneous detection of *S*. Pullorum and *S*. Enteritidis, which are common pathogens in the poultry industry.

This study aimed to develop a rapid and simple dual RPA-LFD method targeting the *traJ* gene of *S*. Pullorum and the *Sdf* *Ⅰ* gene of *S*. Enteritidis. The *traJ* gene is located on the virulence plasmid pSPUV of *S*. Pullorum and is a specific target that can be genetically stable [17]. Meanwhile, the *Sdf* *Ⅰ* gene as an internal fragment of the genomic island φSE14, has been used widely as a specific molecular marker for *S*. Enteritidis [18]. Additionally, through the optimization of RPA amplification conditions, a dual RPA-LFD method capable of simultaneously detecting *S*. Pullorum and *S*. Enteritidis was successfully developed. The practicality of the dual RPA-LFD method was further evaluated by testing clinical samples.

## Materials and methods

### Bacterial culture and DNA extraction

S. Pullorum and *S*. Enteritidis isolated from infected chickens were preserved in the laboratory and stored at −80°C in 20% (v/v) glycerol. For recovery, the above strains were cultured overnight in Luria-Bertani (LB) broth at 37°C, and the serotypes of the bacterial strains were confirmed by PCR amplification. According to the manufacturer’s instructions, genomic DNA was extracted from *S*. Pullorum and *S*. Enteritidis using the TaKaRa MiniBEST Bacterial Genomic DNA Extraction Kit (TaKaRa, Shiga, Japan). The concentration and purity of the extracted DNA templates were measured using a NanoDrop (Thermo Fisher Scientific, Shanghai, China). The DNA templates were stored at −20°C until further use.

### Design of the RPA primers and probes

On the basis of previous reports and the use of VISTA Tools for screening, *traJ* and *Sdf Ⅰ* were identified as potential target genes for the differentiation of *S*. Pullorum and *S*. Enteritidis, respectively [17–19]. The nucleic acid sequences of *traJ* and *Sdf Ⅰ* were obtained from the NCBI database and aligned to identify conserved regions. The forward and reverse primers for RPA were designed using Primer Premier 5 software, and 46 ~ 52 nt fragments were selected as probes between the forward and reverse primers.

The reverse primers from the best selected primer pairs for *S*. Pullorum and *S*. Enteritidis were labeled with biotin at the 5′ end. The *S*. Pullorum probe was labeled with 6-carboxyfluorescein (6-FAM) at the 5′ end and a C3 spacer at the 3′ end, while tetrahydrofuran (THF) was used to label a position 32 nt from the 5′ end of the probe. Similarly, the *S*. Enteritidis probe was labeled with digoxin at the 5′ end and a C3 spacer at the 3′ end, with a THF positioned 33 nt from the 5′ end. All primers and probes (Table 1) were synthesized by Shanghai Sangon Biotech Co., Ltd., China.

**Table 1:** Sequences of the RPA primers and probes.

Primer name	Primer sequence (5′ −3′)	Primer length (bp)
SP-RPA-F1	TTTGTTTCGGGAATTGTTCCTGAAAGAAAC	215
SP-RPA-R1	Biotin-CATACGACAATACCATCAGGTTTACTGTAG	
SP-RPA-F2	TTTCCGAATTGATCTCTATAACCGAGATGG	214
SP-RPA-R2	ATAATCCCCAGAGAATCTGGGTTTGTAATT	
SP-RPA-F3	ATTGTTCCTGAAAGAAACTTATAGTGCTGAAA	249
SP-RPA-R3	TTGTAATTATTCGTATGCCGTACATGAGTTG	
SE-RPA-F1	CCGGGAGAGGCGGTTTGATGTGGTTGGTTCG	205
SE-RPA-R1	Biotin-AGGTGGTGGCTGGCGAATGGTGAGCAGACAA	
SE-RPA-F2	CCGCCGGGAGAGGCGGTTTGATGTGGTTGGT	212
SE-RPA-R2	TCGAAGGTGGTGGCTGGCGAATGGTGAGCAG	
SE-RPA-F3	GAGCATGTTCTGGAAAGCCTCTTTATATAG	194
SE-RPA-R3	ATCTAATGAACTACGTTCGTTCTTCTGGTA	
SP-RPA-Probe	6-FAM-ATATCGAAATGAACATACTCATCTAAATCTT[THF] TTGAGGATATTTTTAT-C3 Spacer	48
SE-RPA-Probe	Dig-GTTCTGGAAAGCCTCTTTATATAGCTCATTCT[THF] ACCTCTAAGCCGGTCAATGA-C3 Spacer	53

### Single RPA basal primer screening

The RPA reaction system was prepared in accordance with the manufacturer’s instructions for the DNA Isothermal Rapid Amplification Kit (Colloidal Gold Test Strip Type) (Amplification Future, Changzhou, China), using three pairs of *S*. Pullorum RPA primers and three pairs of *S*. Enteritidis RPA primers that were specifically designed and listed in Table 1. The total reaction volume was 50 μL, containing 2.3 μL each of the forward and reverse primers. DNA templates of *S*. Pullorum and *S*. Enteritidis were prepared by 10-fold serial dilution. After mixing and centrifugation, the reaction was performed at a constant temperature for 15 min. Electrophoresis was performed on a 1.5% agarose gel, and the optimal primer pairs for *S*. Pullorum and *S*. Enteritidis were selected for subsequent condition optimization.

### Optimization of the dual RPA-LFD method conditions

The dual RPA reaction was performed using a DNA Isothermal Rapid Amplification Kit (Colloidal Gold Test Strip Type) (Amplification Future, Changzhou, China) according to the manufacturer’s instructions. Through preliminary single-tube experiments, the dual RPA method was developed with the best screened primer pairs, and the results were observed using NA Testing 2 (LFD) (Sangon, Shanghai, China). The results were determined by observing the C line and T line of the LFD. The C line is blue and located in the quality control area, whereas the T line is red and located in the detection area. The result of detection was deemed positive if both the C line and the T line appeared and negative if only the C line appeared.

In the experiment, the amplification efficiency among different targets was inconsistent. The optimal probe ratio for *S*. Pullorum and *S*. Enteritidis was determined to achieve similar amplification efficiencies for both fragments. With the total volume of the *S*. Pullorum and *S*. Enteritidis probe mixture remaining unchanged, the SP-RPA-Probe:SE-RPA-Probe ratios tested in the reaction were 9:1, 8:2, 7:3, 6:4, 5:5, 4:6, 3:7, 2:8, and 1:9 to determine the optimal probe ratio. The reaction was performed at 39°C for 15 min. After the reaction, the products were diluted 20-fold, and 80 μL of each diluted product was added to the NA Testing 2 (LFD). The results were interpreted within 5 min after the C line appeared. Using the optimized probe ratio, the optimal reaction temperature and reaction time for dual RPA were also investigated. The dual RPA reaction was performed at 23°C, 28°C, 32°C, 35°C, 37°C, 38°C, 39°C, 41°C, 44°C, 48°C, and 53°C to determine the optimal reaction temperature. Then, the dual RPA reaction was performed at 10 min, 15 min, 18 min, 20 min, 22 min, 25 min, and 30 min to determine the optimal reaction time. All the diluted reaction products were observed with the NA Testing 2 (LFD).

### Assessment of the sensitivity and specificity of the dual RPA-LFD method

To evaluate the sensitivity of the dual RPA-LFD method, we serially diluted the DNA templates of *S*. Pullorum and *S*. Enteritidis 10-fold, with the concentration range of *S*. Pullorum from 1.56 × 10^8^ CFU/mL to 1.56 × 1^00^ CFU/mL and the concentration range of *S*. Enteritidis from 1.38 × 10^8^ CFU/mL to 1.38 × 1^00^ CFU/mL. The nucleic acids of 23 common pathogenic bacterial and viral strains were extracted separately to evaluate the specificity of the dual RPA-LFD method (S1 Table). All the strains were isolated and stored in the laboratory. All of the resulting products were tested with the NA Testing 2 (LFD) for detection purposes.

### Interference assessment of the dual RPA-LFD method

Under clinical conditions, the concentrations of *S*. Pullorum and *S*. Enteritidis in the tested samples were unknown. To evaluate the interference between higher and lower concentrations of nucleic acid templates, we prepared seven artificially coinfected samples with different concentrations of *S*. Pullorum and *S*. Enteritidis, which were analyzed using the dual RPA-LFD method.

### Validation of the dual RPA-LFD method with clinical samples

To evaluate its clinical applicability, the dual RPA-LFD method was used to analyze 50 tissue samples collected from multiple poultry farms in Guangxi, China. The results were subsequently compared with those obtained using the Bacteriological Analytical Manual (BAM) method established by the U.S. Food and Drug Administration.

## Results

### Primer screening

**Fig 1:**
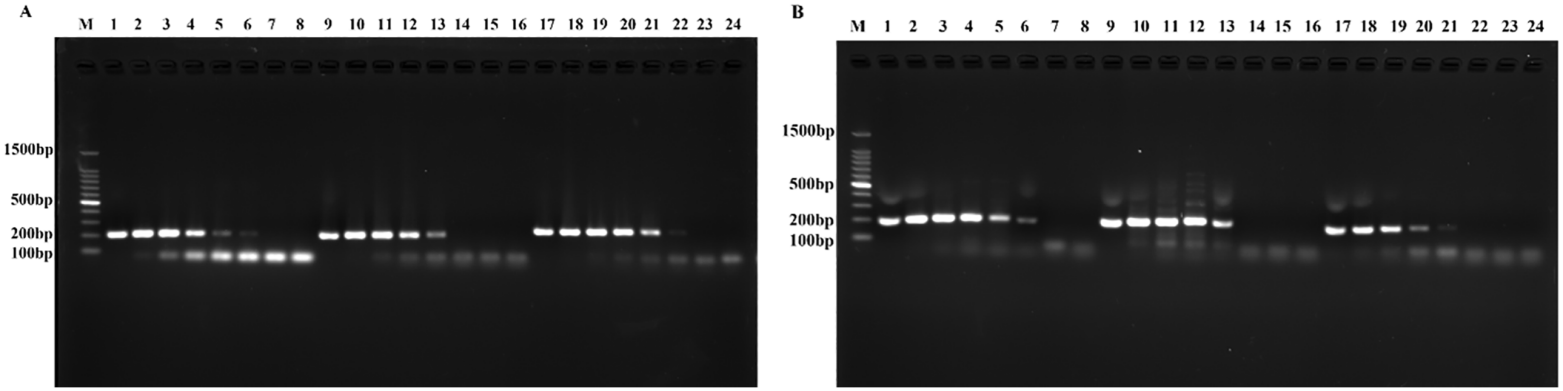
Primer screening. **(A)** Primer screening for ***S*.** Pullorum. M: DNA Ladder; 1–7: SP-RPA-F1/R1; 9–15: SP-RPA-F2/R2; 17–23: SP-RPA-F3/R3; 8, 16, 24: negative control. **(B)** Primer screening for ***S*.** Enteritidis. M: DNA Ladder; 1–7: SE-RPA-F1/R1; 9–15: SE-RPA-F2/R2; 17–23: SE-RPA-F3/R3; 8, 16, 24: negative control.

Three primer pairs each were designed for the *traJ* gene of *S*. Pullorum and the *Sdf* *Ⅰ* gene of *S*. Enteritidis. In primer‒probe combination negative testing and conventional PCR screening, the primer pairs SP-RPA-F1/R1, SP-RPA-F3/R3 and SE-RPA-F1/R1 demonstrated the best specificity and amplification efficiency (Fig 1). Following probe addition, detection using the LFD revealed that the amplification efficiency of SP-RPA-F1/R1 was greater than that of SP-RPA-F3/R3 (S1 Fig). Therefore, the primer pairs SP-RPA-F1/R1 and SE-RPA-F1/R1 were selected for subsequent development of the dual RPA-LFD method.

### Optimization of the reaction conditions for the dual RPA-LFD method

**Fig 2:**
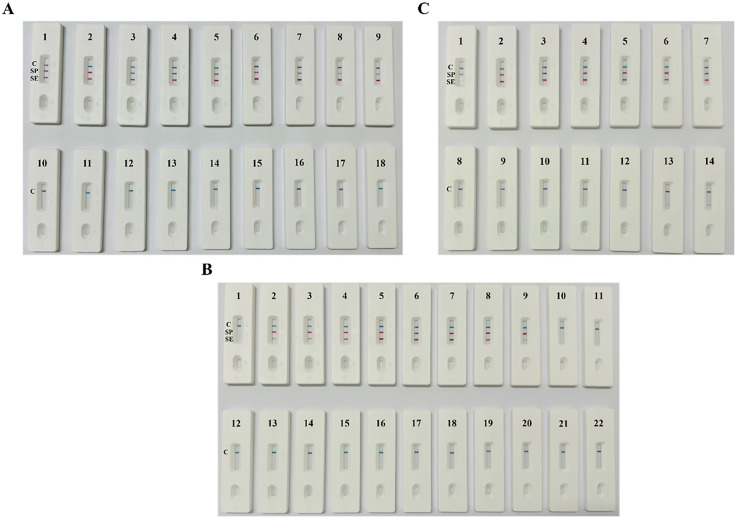
Results of reaction condition optimization. **(A)** Optimization of the probe ratio (1–9: SP-RPA-Probe:SE-RPA-Probe ratios of 9:1, 8:2, 7:3, 6:4, 5:5, 4:6, 3:7, 2:8, and 1:9; 10–18: negative control). **(B)** Optimization of the reaction temperature (1–11: 23°C, 28°C, 32°C, 35°C, 37°C, 38°C, 39°C, 41°C, 44°C, 48°C, and 53°C; 12–22: negative control). **(C)** Optimization of the reaction time (1–7: 10 min, 15 min, 18 min, 20 min, 22 min, 25 min, and 30 min; 8–14: negative control).

Nine different probe ratios and primer combinations were used to determine the optimal probe ratio for *S*. Pullorum and *S*. Enteritidis. All nine groups showed successful amplification of the target bands for both *S*. Pullorum and *S*. Enteritidis, whereas the negative control exhibited a clear C line with no T line observed (Fig 2A). When the probe ratio reached 6:4, the amplification rate tended to balance, and the amplification efficiency peaked.

At the optimal probe ratio, dual RPA-LFD reactions at different temperatures were performed. The results revealed that the T lines at temperatures ranging from 37°C to 41°C were darker, indicating higher reaction efficiency, whereas only the C lines were observed in the negative controls (Fig 2B). To achieve rapid clinical diagnosis, an optimal reaction temperature of 37°C was selected, which corresponds to the typical human body temperature. To determine the optimal reaction time, dual RPA-LFD reactions were performed at a constant temperature of 37°C for different reaction times. Two clear and uniform T lines became visible within 15 min (Fig 2C). However, as the reaction time increased to 22 min, the T line did not significantly darken. When the reaction time was 25 min and 30 min, the negative control gradually showed a weak T line. Therefore, 15 min was determined to be the optimal reaction time.

### Sensitivity and specificity of the dual RPA-LFD method

**Fig 3:**
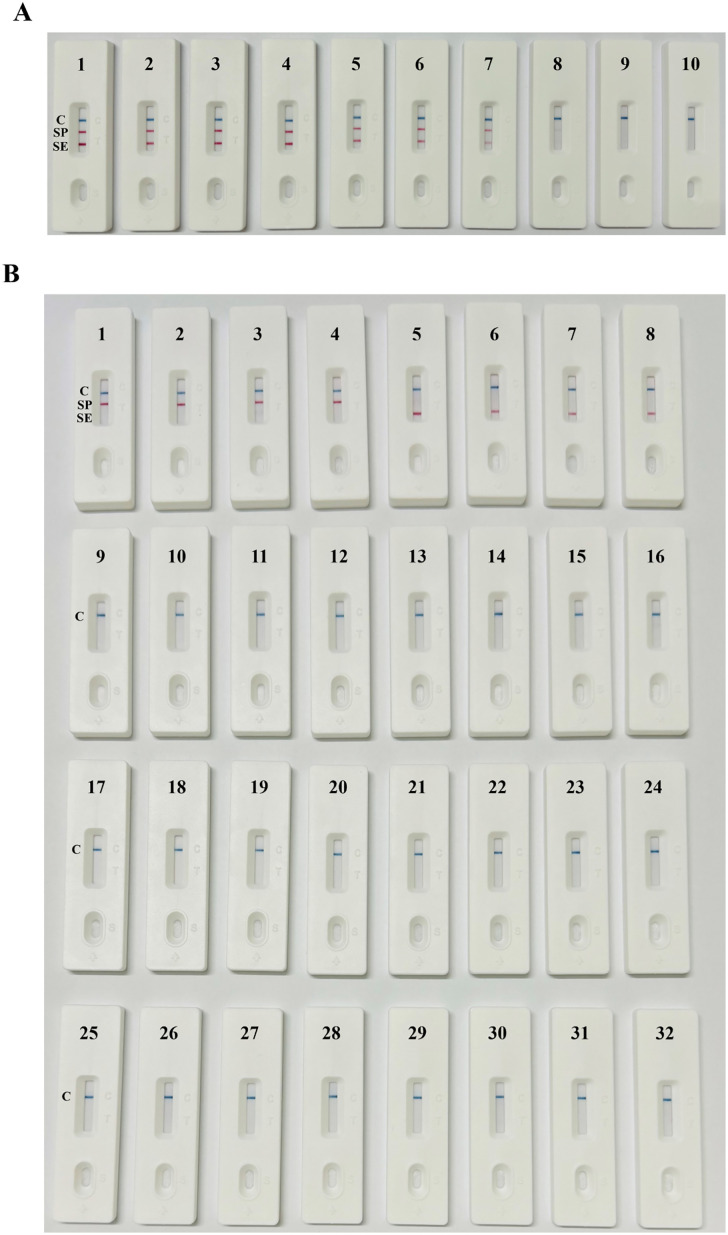
Results of the sensitivity and specificity tests. **(A)** Sensitivity of the dual RPA-LFD method. 1–9 (*S*. Pullorum/*S*. Enteritidis): 1.56/1.38 × 10^8^ CFU/mL, 1.56/1.38 × 10^7^ CFU/mL, 1.56/1.38 × 10^6^ CFU/mL, 1.56/1.38 × 10^5^ CFU/mL, 1.56/1.38 × 10^4^ CFU/mL, 1.56/1.38 × 10^3^ CFU/mL, 1.56/1.38 × 10^2^ CFU/mL, 1.56/1.38 × 10^1^ CFU/mL, and 1.56/1.38 × 10^0^ CFU/mL; 10: negative control. **(B)** Specificity of the dual RPA-LFD method (1–4: ***S*.** Pullorum; 5–8: ***S*.** Enteritidis; 9–31: other pathogens; 32: negative control).

To test the sensitivity of the dual RPA-LFD method, 10-fold serial dilutions of purified *S*. Pullorum and *S*. Enteritidis genomic DNA were used as templates. The highest concentrations of the *S*. Pullorum and *S*. Enteritidis templates were 1.56 × 10^8^ CFU/mL and 1.38 × 10^8^ CFU/mL, respectively, and the T line was invisible to the naked eye when the concentration was below 10^2^ CFU/mL (Fig 3A). Therefore, the detection limits for the dual RPA-LFD method to detect *S*. Pullorum and *S*. Enteritidis were 1.56 × 10^2^ CFU/mL and 1.38 × 10^2^ CFU/mL, respectively.

The nucleic acids of *S*. Pullorum, *S*. Enteritidis, and other common pathogens were used for specificity determination via the dual RPA-LFD method. The results revealed that a clear C line and T line could be observed on the LFD with *S*. Pullorum and *S*. Enteritidis nucleic acids, whereas only the C line could be observed for the other pathogens (Fig 3B). Furthermore, the dual RPA-LFD method could accurately distinguish *S*. Pullorum, *S*. Enteritidis, and other common *Salmonella* serotypes, indicating that the method has high specificity.

### Interference in the dual RPA-LFD method

**Fig 4:**
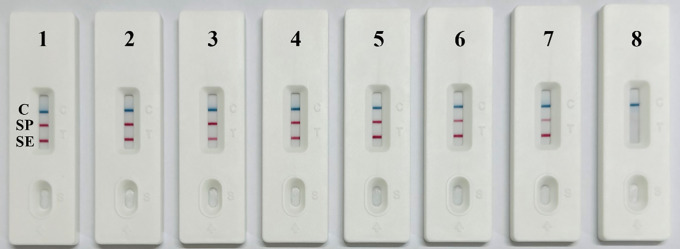
Results of interference experiments. 1: SP-1.56 × 10^6^ CFU/mL, SE-1.38 × 10^5^ CFU/mL; 2: SP-1.56 × 10^6^ CFU/mL, SE-1.38 × 10^4^ CFU/mL; 3: SP-1.56 × 10^6^ CFU/mL, SE-1.38 × 10^3^ CFU/mL; 4: SP-1.56 × 10^6^ CFU/mL, SE-1.38 × 10^6^ CFU/mL; 5: SP-1.56 × 10^5^ CFU/mL, SE-1.38 × 10^6^ CFU/mL; 6: SP-1.56 × 10^4^ CFU/mL, SE-1.38 × 10^6^ CFU/mL; 7: SP-1.56 × 10^3^ CFU/mL, SE-1.38 × 10^6^ CFU/mL; 8: negative control.

The results of the dual RPA-LFD interference experiments revealed that the two T lines could still be observed simultaneously when the DNA concentration ratio of *S*. Pullorum and *S*. Enteritidis was approximately 1000 (Fig 4). These results demonstrated that in the dual RPA-LFD method, DNA templates at higher concentrations did not inhibit the amplification of DNA templates at lower concentrations, and vice versa.

### Results of clinical sample testing

*S*. Pullorum and *S*. Enteritidis were detected in 50 clinical tissue samples using the developed dual RPA-LFD method. The results revealed that 13 samples were positive for *S*. Pullorum, 6 samples were positive for *S*. Enteritidis, and 2 samples were positive for both pathogens (Table 2). These findings were consistent with the positivity rates observed by the BAM method.

**Table 2:** Clinical sample detection.

Pathogens	Dual RPA-LFD	BAM
*S*. Pullorum	13 (26%)	13 (26%)
*S*. Enteritidis	6 (12%)	6 (12%)
*S*. Pullorum+*S*. Enteritidis	2 (4%)	2 (4%)
Negative	29 (58%)	29 (58%)
Total	50	50

## Discussion

*S*. Pullorum is a host-specific pathogen that primarily causes disease and death in chickens through vertical transmission, and large-scale poultry farms prevent and control *S*. Pullorum infection through breeding stock purification [6]. *S*. Enteritidis is a multihost pathogen and the predominant serotype on poultry farms where *S*. Pullorum has been eradicated [20]. *S*. Enteritidis can infect chickens through both vertical and horizontal transmission and is difficult to eliminate through breeding stock purification programs [21]. Both *S*. Pullorum and *S*. Enteritidis pose substantial threats to the poultry industry in practical breeding processes. Currently, *Salmonella* detection methods predominantly rely on BAM and PCR [2]. However, BAM is time-consuming and laborious, and the slide agglutination test in BAM is prone to false-positive results. PCR requires complex instrumentation and well-equipped laboratories. Neither of these methods is suitable for onsite testing, such as on farms or in the clinic.

In recent years, with the emergence of isothermal amplification methods, RPA has been widely used as a representative technique for nucleic acid detection for various pathogens [22–24]. Compared with other methods, RPA has significant advantages, such as short amplification times, low temperatures, low cost and strong specificity for the sample [25]. In this study, a dual RPA-LFD method for *S*. Pullorum and *S*. Enteritidis was successfully developed through the combination of RPA and LFD. The primers used were designed to target the *traJ* gene of *S*. Pullorum and the *Sdf* *Ⅰ* gene of *S*. Enteritidis. The *traJ* gene is specific to and genetically stable in *S*. Pullorum [17,26]. The *Sdf* *Ⅰ* gene is highly specific and sensitive to *S*. Enteritidis [27]. To achieve double detection, all reverse primers were labeled with biotin at the 5′ end; the *S*. Pullorum and *S*. Enteritidis probes were labeled with 6-FAM and digoxin at the 5′ end, respectively. After the RPA reaction was complete, amplicons with 6-FAM and biotin moieties, and digoxin and biotin moieties were generated respectively. Then, an LFD with two test lines was prepared for the detection of dual RPA amplification products. The 6-FAM and biotin moieties generated by the *S*. Pullorum reaction bound to the anti-FAM antibody on the T1 line of the LFD, whereas the digoxin and biotin moieties generated by the *S*. Enteritidis reaction bound to the anti-digoxin antibody on the T2 line of the LFD.

In this study, the dual RPA-LFD method had a high amplification efficiency within a broad isothermal range of 37 ~ 41°C. This reaction temperature range does not require professional thermal cycling equipment, allowing the use of even body heat-based techniques such as armpit incubation, making it suitable for field tests in resource-limited areas [28]. The optimal reaction time for this dual RPA method was 15 min, which was shorter by more than 5 min than that of the SP-RPA-LFNAA method established by Liu and the SE-PA-LAMP method established by Lamas [29,30]. When the reaction time was extended to 25 min and 30 min, a T line gradually appeared in the negative control, indicating that prolonged incubation durations could promote the generation of non-specific byproducts such as primer dimers, which might lead to false-positive results. Hence, it is critical to strictly control the reaction time in practical applications. The detection limits for *S*. Pullorum and *S*. Enteritidis were 1.56 × 10^2^ CFU/mL and 1.38 × 10^2^ CFU/mL, respectively. Notably, differences in template concentration did not interfere with the experimental results. This method enables the simultaneous identification of both pathogens in a single test, meeting the requirements for rapid onsite detection of bacterium-contaminated samples.

The developed dual RPA-LFD method is highly specific, showing no cross-amplification not only between the two target bacteria but also between other common bacteria and viruses. Additionally, compared with the BAM method, this method demonstrated consistent detection of *S*. Pullorum and *S*. Enteritidis in positive samples. These results confirm the accuracy of the test and indicate that the dual RPA-LFD method has practical application value. Furthermore, like most other nucleic acid amplification techniques, RPA is also susceptible to residual contamination, especially when the test tube is opened after amplification to observe the results [31]. To avoid aerosol contamination of reaction products, in practical applications, the product should be diluted, and the results should be observed in a well-ventilated outdoor area.

In conclusion, the dual RPA-LFD method for *S*. Pullorum and *S*. Enteritidis developed in this study has the advantages of specificity, sensitivity, time efficiency and convenience. This method has potential application value in resource-limited areas and primary veterinary laboratories.

## Supporting information

S1 Fig Primer amplification efficiency. (A) Amplification efficiency of SP-RPA-F1/R1: 1–8 (*S*. Pullorum): 1.56 × 10^8^ CFU/mL, 1.56 × 10^7^ CFU/mL, 1.56 × 10^6^ CFU/mL, 1.56 × 10^5^ CFU/mL, 1.56 × 10^4^ CFU/mL, 1.56 × 10^3^ CFU/mL, 1.56 × 10^2^ CFU/mL, and 1.56 × 10^1^ CFU/mL; 9: negative control. (B) Amplification efficiency of SP-RPA-F3/R3: 1–7 (*S*. Pullorum): 1.56 × 10^8^ CFU/mL, 1.56 × 10^7^ CFU/mL, 1.56 × 10^6^ CFU/mL, 1.56 × 10^5^ CFU/mL, 1.56 × 10^4^ CFU/mL, 1.56 × 10^3^ CFU/mL, and 1.56 × 10^2^ CFU/mL; 8: negative control.
(DOCX)

S1 Table Information on the bacterial and viral strains used for the specificity tests. (DOCX)
