# Supplementary material for: Establishment and application of a dual RPA-LFD rapid detection method for Salmonella Pullorum and Salmonella Enteritidis
Source: PLoS One. 2025 Nov 19;20(11):e0336423. doi: 10.1371/journal.pone.0336423 (PMC12629420; doi:10.1371/journal.pone.0336423)
Supplement: S1 Fig — (A) Amplification efficiency of SP-RPA-F1/R1: 1–8 (S. Pullorum): 1.56 × 10^8^ CFU/mL, 1.56 × 10^7^ CFU/mL, 1.56 × 10^6^ CFU/mL, 1.56 × 10^5^ CFU/mL, 1.56 × 10^4^ CFU/mL, 1.56 × 10^3^ CFU/mL, 1.56 × 10^2^ CFU/mL, and 1.56 × 101 CFU/mL; 9: negative control. (B) Amplification efficiency of SP-RPA-F3/R3: 1–7 (S. Pullorum): 1.56 × 10^8^ CFU/mL, 1.56 × 10^7^ CFU/mL, 1.56 × 10^6^ CFU/mL, 1.56 × 10^5^ CFU/mL, 1.56 × 10^4^ CFU/mL, 1.56 × 10^3^ CFU/mL, and 1.56 × 10^2^ CFU/mL; 8: negative control. (DOCX) [file pone.0336423.s001.docx]

**
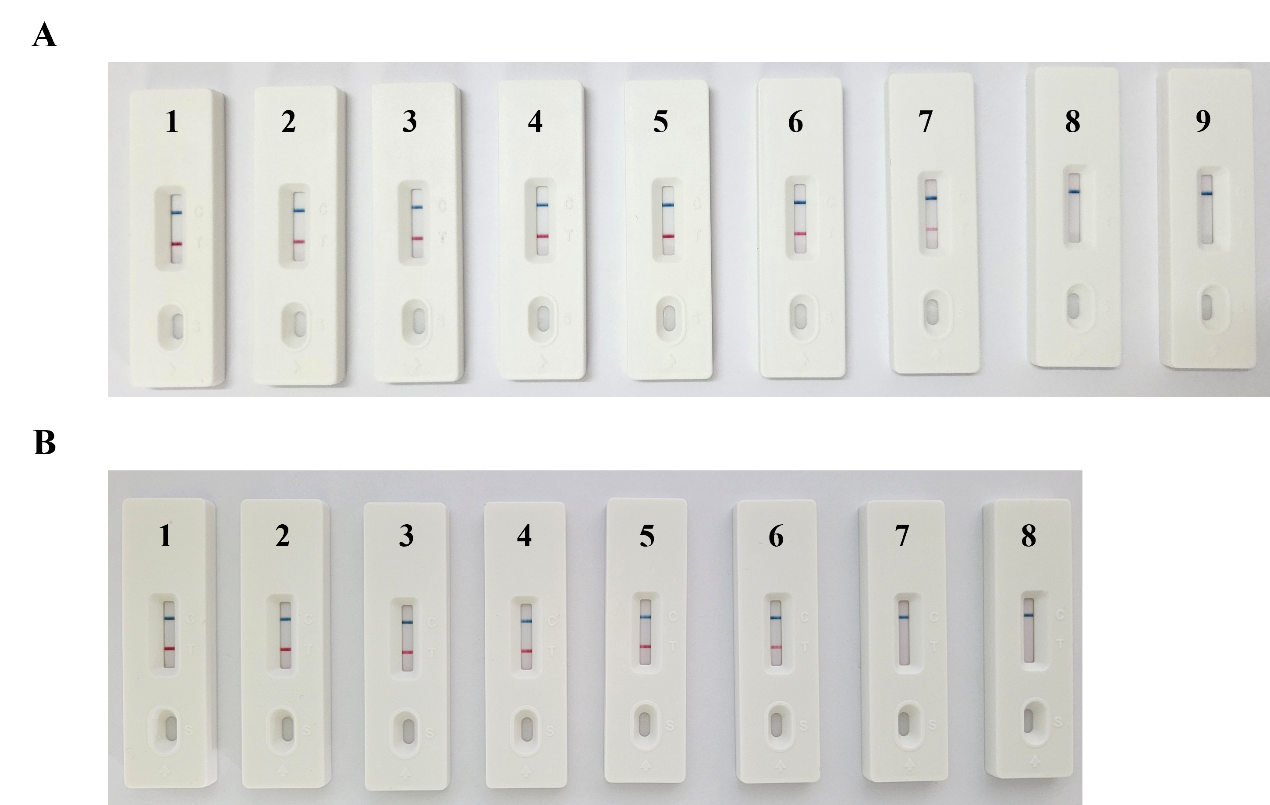
**

**S1 Fig. Primer amplification efficiency.** (A) Amplification efficiency of SP-RPA-F1/R1: 1-8 (*S*. Pullorum): **1.56** × 10^8^ CFU/mL, **1.56** × 10^7^ CFU/mL, **1.56** × 10^6^ CFU/mL, **1.56** × 10^5^ CFU/mL, **1.56** × 10^4^ CFU/mL, **1.56** × 10^3^ CFU/mL, **1.56** × 10^2^ CFU/mL, **and 1.56** × 10^1^ CFU/mL; **9: negative control.** (B) Amplification efficiency of SP-RPA-F3/R3: 1-7 (*S*. Pullorum): **1.56** × 10^8^ CFU/mL, **1.56** × 10^7^ CFU/mL, **1.56** × 10^6^ CFU/mL, **1.56** × 10^5^ CFU/mL, **1.56** × 10^4^ CFU/mL, **1.56** × 10^3^ CFU/mL, **and 1.56** × 10^2^ CFU/mL; **8: negative control.**
