# Supplementary material for: Establishment and application of a dual RPA-LFD rapid detection method for Salmonella Pullorum and Salmonella Enteritidis
Source: PLoS One. 2025 Nov 19;20(11):e0336423. doi: 10.1371/journal.pone.0336423 (PMC12629420; doi:10.1371/journal.pone.0336423)
Supplement: S1 Table — (DOCX) [file pone.0336423.s002.docx]

**S1** **Table.** **Information on the bacterial and viral strains used for the specificity tests.**

| Serial Number | Species | Strain name |
| --- | --- | --- |
| 1 | *Salmonella* Pullorum | Chicken/nanning/2024L210 |
| 2 | *Salmonella* Enteritidis | Chicken/nanning/2024D40 |
| 3 | *Salmonella* Typhimurium | Chicken/nanning/20160417 |
| 4 | *Salmonella* Derby | Chicken/nanning/20160920 |
| 5 | *Salmonella* Kentucky | Chicken/nanning/20170112 |
| 6 | *Salmonella* Infantis | Chicken/nanning/20160306 |
| 7 | *Salmonella* Gallinarum | Chicken/nanning/20170824 |
| 8 | *Escherichia coli* | Chicken/nanning/20160223 |
| 9 | *Staphylococcus aureus* | Chicken/nanning/A56 |
| 10 | *Listeria monocytogenes* | L1 |
| 11 | *Pasteurella* | P3 |
| 12 | *Campylobacter jejuni* | CICC 22936 |
| 13 | *Mycoplasma gallisepticum* (MG) | S6 |
| 14 | *Mycoplasma synoviae* (MS) | PMS-256 |
| 15 | Reticuloendotheliosis virus (REV) | REV-T |
| 16 | Chicken Infectious Anemia Virus (CIAV) | CIAV-1 |
| 17 | Fowl Adenovirus (FAdV) | GX001 |
| 18 | Infectious Bronchitis Virus (IBV) | H120 |
| 19 | Infectious Laryngotracheitis Virus (ILTV) | Beijing strain |
| 20 | Avian Reovirus (ARV) | S1133 |
| 21 | Marek’s Disease Virus (MDV) | CVI988 |
| 22 | Avian Influenza Virus H5 | Duck/Guangxi/1/04 |
| Serial Number | Species | Strain name |
| 23 | Avian Influenza Virus H7 | A/Duck/42846/07 |
| 24 | Avian Influenza Virus H9 | A/Guangxi/020B7/2010 |
| 25 | Duck Hepatitis Virus (DHV) | DHV-1 |
